# Formulation of Nanomicelles to Improve the Solubility and the Oral Absorption of Silymarin

**DOI:** 10.3390/molecules24091688

**Published:** 2019-04-30

**Authors:** Vieri Piazzini, Mario D’Ambrosio, Cristina Luceri, Lorenzo Cinci, Elisa Landucci, Anna Rita Bilia, Maria Camilla Bergonzi

**Affiliations:** 1 Department of Chemistry, University of Florence, Via U. Schiff 6, 50019 Sesto Fiorentino, Florence, Italy; vieri.piazzini@unifi.it (V.P.); ar.bilia@unifi.it (A.R.B.); 2 Department of Neurosciences, Psychology, Drug Research and Child Health (NEUROFARBA), Section of Pharmacology and Toxicology, University of Florence, Viale Pieraccini 6, 50139 Florence, Italy; mario.dambrosio@unifi.it (M.D.); cristina.luceri@unifi.it (C.L.); lorenzo.cinci@unifi.it (L.C.); 3 Department of Health Sciences, Section of Clinical Pharmacology and Oncology, University of Florence, Viale Pieraccini 6, 50139 Florence, Italy; elisa.landucci@unifi.it

**Keywords:** silymarin, drug delivery, polymeric nanomicelles, mixed nanomicelles, antioxidant activity, PAMPA, Caco-2 cell line, oral bioavailability

## Abstract

Two novel nanomicellar formulations were developed to improve the poor aqueous solubility and the oral absorption of silymarin. Polymeric nanomicelles made of Soluplus and mixed nanomicelles combining Soluplus with d-α-tocopherol polyethylene glycol 1000 succinate (vitamin E TPGS) were prepared using the thin film method. Physicochemical parameters were investigated, in particular the average diameter, the homogeneity (expressed as polydispersity index), the zeta potential, the morphology, the encapsulation efficiency, the drug loading, the critical micellar concentration and the cloud point. The sizes of ~60 nm, the narrow size distribution (polydispersity index ≤0.1) and the encapsulation efficiency >92% indicated the high affinity between silymarin and the core of the nanomicelles. Solubility studies demonstrated that the solubility of silymarin increased by ~6-fold when loaded into nanomicelles. Furthermore, the physical and chemical parameters of SLM-loaded formulations stored at room temperature and in refrigerated conditions (4 °C) were monitored over three months. In vitro stability and release studies in media miming the physiological conditions were also performed. In addition, both formulations did not alter the antioxidant properties of silymarin as evidenced by the 1,1-Diphenyl-2-picrylhydrazyl radical (DPPH) assay. The potential of the nanomicelles to increase the intestinal absorption of silymarin was firstly investigated by the parallel artificial membrane permeability assay. Subsequently, transport studies employing Caco-2 cell line demonstrated that mixed nanomicelles statistically enhanced the permeability of silymarin compared to polymeric nanomicelles and unformulated extract. Finally, the uptake studies indicated that both nanomicellar formulations entered into Caco-2 cells via energy-dependent mechanisms.

## 1. Introduction

Silymarin (SLM) is a mixture of flavonolignans (silybin A and silybin B, isosilybin A and isosilybin B, silychristin A and silychristin B, isosilychristin A and isosilychristin B, silydianin, silymonin, cisilybin A and cisilybin B, isocisilybin A and isocisilybin B, silandrin A and silandrin B, cisilandrin A and cisilandrin B, isosilandrin A and isosilandrin B, isocisilandrin A and isocisilandrin B, silyhermin A and silyhermin B, neosilyhermin A and neosilyhermin B), flavonoids (taxifolin and quercetin), fatty acids, proteins, fixed oil, betaine and polyphenols extracted from fruits of *Silybum marianum* (L.) Gaertn. (asteraceae), also known as milk thistle [1]. Since SLM can induce the regeneration of hepatocytes, it has been used since the ancient times in the treatment of liver and gallbladder diseases [2,3]. Moreover, SLM has antioxidant and anti-inflammatory properties, and its efficacy in the treatment of metabolic disorders in diabetes was reported, in particular with regard to lipid profile and blood glucose level [4], cancer [5], neurological disorders [6], cardiac [7], gastrointestinal [8], lung [9], skin [10] and renal diseases [11]. In addition, the benefits of SLM against radiotherapy-induced mucositis and hand-foot syndrome in patients treated with capecitabine are well documented [12,13].

However, the aqueous solubility of SLM is poor, and it is usually administered in adult patients in the form of capsules at a dosage of 240–800 mg/day [14,15]. Moreover, pharmacokinetic analysis revealed that after oral administration to human healthy volunteers the main flavonolignans of SLM (silybin A, silybin B, isosilybin A, isosilybin B, silychristin and silydianin) are metabolized to their conjugates (sulfates and glucuronides) and rapidly eliminated with relatively short half-lives (1–3, 3–6, and 3–5 h for the free, conjugated and total SLM flavonolignans, respectively) [16].

To overcome these drawbacks, in particular the low aqueous solubility and limited oral bioavailability, several strategies were employed in recent years, including complexation with phospholipids (phytosomes) [17], inclusion complex with β-cyclodextrins [18], solid dispersions [19], microparticles [20], polymeric nanoparticles [21], liposomes [22], solid lipid nanoparticles [23], nanostructured lipid carriers [24], micro-/nanoemulsions [25,26], self-microemulsifying drug delivery systems [27] and polymeric micelles [28,29].

In the last few years, nanomicelles have gained increasing attention in the diagnosis and treatments of many pathologies, culminated with the approval by the Food and Drug Administration (FDA) of Genexol^®^PM, a micelle formulation of paclitaxel for the treatment of breast, ovarian and lung cancer in 2007 [30]. Nanomicelles are generally made of amphiphilic polymers that self-assemble in water into hydrophobic core-hydrophilic shell nanostructures (20–200 nm) at concentrations higher than the critical micellar concentration (CMC). The presence of the lipophilic core increases the solubility of poorly water-soluble molecules and offers the possibility to obtain a controlled drug release [31], while the hydrophilic shell protects the encapsulated drug from the external medium and prevents the interaction with plasma components, resulting in long circulation properties in vivo. Moreover, the small particle size prolongs the residence time in blood circulation, bypassing the liver and spleen filtration and the glomerular elimination, and enhances cellular uptake and the ability to cross epithelial barriers. All these aspects result in increased drug bioavailability [32].

Hereby, the aim of the present study was to investigate and compare polymeric nanomicelles (PNM) and mixed nanomicelles (MNM) as oral dosage forms to enhance the solubility and the intestinal absorption of SLM. Soluplus was employed as amphiphilic polymer for the development of PNM. Many researchers reported its ability to improve the oral bioavailability of poorly water-soluble drugs [33,34]. In addition, it exerts inhibitory activity on P-glycoprotein (P-gp) efflux pumps [35]. In this regard, considering that SLM is a P-gp substrate [36], MNM were also developed combining Soluplus with d-α-tocopherol polyethylene glycol 1000 succinate, also known as vitamin E TPGS (TPGS). TPGS is widely employed in the food and drug industry as an emulsifier, stabilizer, solubilizer and permeation enhancer. In addition, TPGS has been used to enhance the stability of nano-drug delivery systems [37] and to inhibit the P-gp-mediated efflux enhancing the drug absorption in the intestinal lumen [38]. To the best of our knowledge, there are no reports in which SLM was formulated into Soluplus and Soluplus/TPGS nanomicelles, and for the first time the influence of these excipients on SLM permeation and P-gp-mediated efflux in Caco-2 cell line was studied. Empty and SLM-loaded PNM and MNM were chemically and physically characterized in terms of size, homogeneity, zeta potential, morphology, CMC, cloud point, encapsulation efficiency, loading capacity and storage stability. Moreover, the antioxidant properties of unformulated SLM and SLM-loaded nanomicelles were compared. In vitro stability studies in simulated gastrointestinal environment and blood conditions were performed. The ability of PMN and MNM to improve the passive permeation of SLM was first evaluated by using the Parallel Artificial Membrane Permeability Assay (PAMPA), then by employing the Caco-2 cells.

## 2. Results and Discussion

### 2.1. Preparation and Characterization of Nanomicelles

In this work, the thin film method was used to prepare nanomicelles formulations [39]. Since a low CMC value indicates a high resistance of nanomicelles against dilution by body fluids [40], the total polymer concentration (5% *w*/*v*) was selected above the CMC value of the surfactant(s). Soluplus consists of polyvinyl caprolactam (57%), polyvinyl acetate (30%) and polyethylene glycol (13%). It has a very low CMC value (7.6 mg/L) that confers stability to micellar formulations upon dilution in vivo, and it is considered a safe excipient since no adverse effects were observed in animals at a dose of 1000 mg/Kg [33,34,35]. TPGS is a PEGylated vitamin E with amphiphilic properties and a relatively low CMC (0.02% *w*/*w*). Furthermore, it is recognized by the FDA as a safe pharmaceutic excipient [37]. SLM-PNM were prepared testing increasing amounts of extract powder, from 0.5 mg/mL to 4 mg/mL. PNM loaded with 0.5, 1, 2, 3 mg/mL of SLM had similar average diameter and homogeneity to the empty PNM, while in presence of 4 mg/mL of the extract, the PNM were larger (>200 nm) and highly polydispersed (Polydispersity Index, PdI > 0.3). Thus, 3 mg/mL (corresponding to 1.63 mg/mL of SLM) was selected as the optimal extract concentration (Table 1).

For the optimization of SLM-MNM, different Soluplus/TPGS gravimetric ratios were tested (from 20:1 to 2:1). As the concentration of TPGS increased, the average diameter and the PdI of SLM-MNM increased correspondingly. Since an average hydrodynamic diameter below 100 nm can increase the intestinal drug absorption of nanomicelles after oral administration [33], only SLM-MNM obtained with Soluplus/TPGS 20:1 showed the appropriate technological parameters (Table 1).

**Figure 1:**
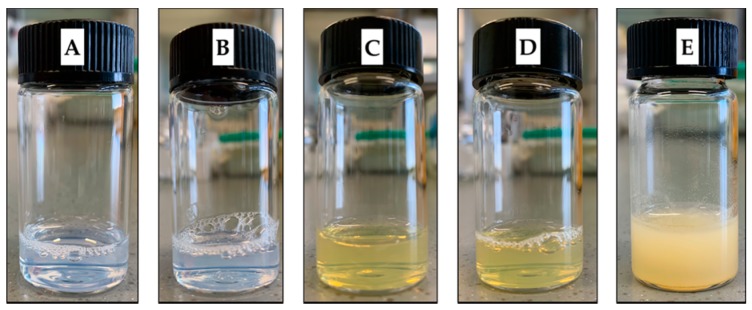
Visual appearance of the nanomicelles. (**A**) Empty polymeric nanomicelles (PNM); (**B**) Empty mixed nanomicelles (MNM); (**C**) Silymarin (SLM)-loaded PNM; (**D**) SLM-loaded MNM; (**E**) SLM aqueous suspension.

Empty PNM and MNM had a light-blue appearance, while SLM-loaded formulations were transparent with a light-yellow opalescence (Figure 1).

The calculated CMC values of PNM and MNM were 0.3 × 10^−3^ mM and 1.5 × 10^−3^ mM, respectively. By increasing the concentration of TPGS, the CMC value of the system increased. Thus, according to the average diameter and PdI, the ratio of 20:1 Soluplus/TPGS was chosen as the best formulation. The CMC value of the micellar formulations is a crucial parameter because low CMC values ensure stability, resistance against dissociation and prevent the loss of the encapsulated drug during the dilution by the body fluids [41]. In general, a CMC value less than 135 mg/L is considered enough to resist toward dissociation upon dilution after oral administration [40]. The low CMC of the developed PNM and MNM can be attributed to the presence of highly lipophilic regions of the polymers employed.

**Figure 2:**
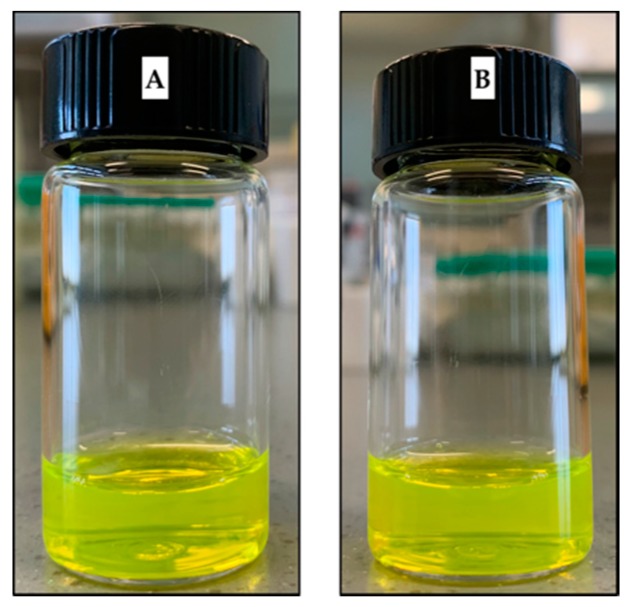
Visual appearance of the fluorescent nanomicelles. (**A**) FITC-loaded PNM; (**B**) FITC-loaded MNM.

Fluorescent PNM and MNM were prepared for in vitro uptake studies adding fluorescein isothiocyanate (FITC) instead of SLM and using the same amounts of Soluplus (for PNM) and Soluplus/TPGS (for MNM) described for empty formulations and SLM-loaded nanomicelles. The results are reported in Table 1. FITC-loaded nanomicelles had an intense yellow-green color (Figure 2).

As displayed in Table 1, all developed formulations showed very small particle size (<100 nm), which could promote the absorption by enterocytes through endocytosis and help to avoid the uptake by the cells of the reticuloendothelial system (RES) and thus bypass the liver and spleen filtration [42]. Both PNM and MNM have a narrow PdI, which was around 0.10. The SLM-loading did not substantially alter the particle size, the homogeneity and the surface charge of PNM and MNM. It is reported that amphiphilic polymers self-assemble into nanostructures in water at a concentration exceeding the CMC. These nanostructures are characterized by a core-shell architecture in which the hydrophobic segments of the polymers form the core, while the hydrophilic chains form the shell. Hydrophobic molecules, such as the flavonolignans of SLM, can be physically incorporated into the core of the nanomicelles mainly via hydrophobic interactions, hydrogen bonds and van der Waals forces [43].

The zeta potential of empty PMN, MNM and SLM/FITC-loaded nanomicelles was neutral. This characteristic may be due to the presence of the non-ionic polymer polyethylene glycol (PEG), in the shell of the micelles [44]. PEG confers stability to the nanomicelles in aqueous media and body fluids, prevents aggregation phenomena and limits the interaction between plasma proteins, avoiding the opsonization process [31]. Moreover, nanoformulations with average diameter below 100 nm that are neutral or negatively charged are effective to penetrate across the anionic mucosal layer at the intestinal level [32].

**Figure 3:**
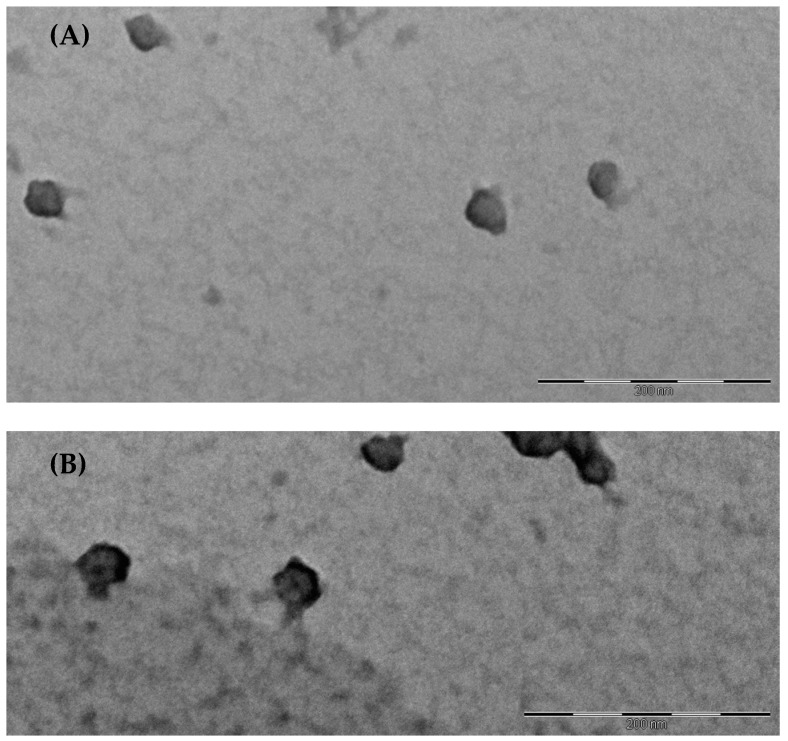
(**A**) Transmission electron microscope (TEM) image of SLM-loaded polymeric nanomicelles (SLM-PNM). Scale bar 200 nm; (**B**) TEM image of SLM-loaded mixed nanomicelles (SLM-MNM). Scale bar 200 nm.

The morphology of SLM-PNM and SLM-MNM was evaluated by transmission electron microscopy (TEM). The images revealed the presence of nanostructures with spherical shape and diameter consistent with DLS data (Figure 3).

Both PNM and MNM exhibited high encapsulation efficiency (EE%) and loading capacity (LC%), indicating that SLM was successfully incorporated into the nanostructures and the high affinity of the extract with the core of the polymers. Indeed, it is known that if the affinity between the drug and the core of the micelles is low, the drug will be not successfully incorporated [44].

### 2.2. Solubilization Capacity Determination

The solubilization capacity of PNM and MNM was investigated adding an excess of SLM to each micellar formulation. To investigate the effect of TPGS on SLM solubility, different gravimetric ratio between Soluplus and TPGS were employed to prepare MNM (Table 2).

As reported in Table 2, all micellar formulations increased the solubility of SLM compared with the aqueous solubility. Soluplus PNM determined the highest improvement of water solubility of the extract (more than 6-fold). The Sf for MNM was lower than PNM, in particular with increasing concentrations of TPGS. This fact might be attributed to the presence of TPGS, which is characterized by a smaller lipophilic portion compared to Soluplus [45]. Thus, these results evidenced that Soluplus played a key role in increasing the solubility of SLM, and the optimal Soluplus/TPGS ratio for MNM was 20:1, according to the physical characterization.

### 2.3. Cloud Point

The cloud point is the temperature at which a homogenous solution of amphiphilic polymers presents a cloudy appearance [46]. The increase in temperature causes the dehydration of the hydrophilic chain of the polymers, resulting in micelles aggregation and loss of the stability of the nanosystem. The determination of the cloud point helps to select the storage conditions and to predict the stability of the formulation after administration. The cloud point of SLM-PNM and SLM-MNM was investigated. SLM-PNM exhibited a cloud point of 40.7 ± 0.8 °C (Mean ± SD, *n* = 3), while SLM-MNM of 38.2 ± 1.5 °C (Mean ± SD, *n* = 3). The slight decrease of cloud point for SLM-MNM might be due to the penetration of TPGS in the core of the nanomicelles that causes water expulsion, resulting in increased hydrophobic interactions [44,45].

### 2.4. Stability Studies

#### 2.4.1. Storage Stability

The chemical and physical storage stability of nanomicelles was monitored over three months both in refrigerated conditions (4 °C) and at room temperature. The physical parameters were evaluated by light scattering analyses, the EE% was determined by HPLC, while the presence of any SLM precipitate was visually checked.

As shown in Table 3 and Table 4, both SLM-PNM and SLM-MNM were stable. The physical and chemical parameters before and after storage were substantially comparable. Moreover, no SLM precipitates were observed in all samples. These results are interesting, since it is reported that micellar formulations composed with different Soluplus/TPGS ratios compared to those proposed in the present work are not stable during the storage for a long time [47].

#### 2.4.2. Gastrointestinal Stability

The main obstacle for oral drug delivery is the harsh environment of the gastrointestinal tract. The dissociation of the nanomicelles in the stomach and/or in the intestine causes the release of the encapsulated drug. On the other hand, particle size plays a key role in the gastrointestinal absorption, and it is reported that an average diameter less than 300 nm is advantageous for intestinal permeation [48]. To simulate the gastrointestinal conditions, SLM-PNM and SLM-MNM were incubated at 37 °C in simulated gastric fluid (GF) followed by simulated intestinal fluid (IF).

As shown in Table 5, the average diameter of both formulations was comparable to that measured before the experiment (Table 1), indicating that neither low pH value nor digestive enzymes influence the stability of the developed nanomicelles. This could be due to the steric stabilization effect of the PEG chains of the polymers [49]. Moreover, no SLM precipitation was found, confirming the stability of both the formulations. Based on these results, it is conceivable that SLM could be absorbed at the gastrointestinal level without the degradation of the carrier.

#### 2.4.3. Stability in Blood Conditions

After assessing the physical stability of nanomicelles in gastrointestinal conditions, the formulations were incubated in phosphate buffer saline (PBS, pH 7.4) without and in presence of human serum albumin (HSA, 45 g/L) at 37 °C for 72 h to simulate the blood circulation.

The data reported in Table 6 suggest that both formulations were unchanged in PBS and in PBS with HSA over a period of 72 h. The slight increase of the PdI after incubation in PBS with HSA might be due to the coexistence of albumin and nanomicelles. The maximal increase of the sizes was about 10–15 nm, therefore, the nanomicelles are able to maintain their structure in physiological pH conditions and also in the presence of plasma proteins.

### 2.5. In Vitro Release Studies

To confirm the hypothesis that SLM-PNM and SLM-MNM are stable in the gastrointestinal tract and blood conditions, the release of SLM was monitored in different pH conditions comparing SLM-loaded nanomicelles and an SLM ethanolic solution. Each medium was supplemented with Tween 80 (0.5% *w*/*v*) to obtain sink conditions [50]. After 2 h in GF, 42.4% ± 0.5 of free-SLM in ethanol was released, 85.7% ± 1.1 of free-SLM was released in IF within 6 h, in PBS more than 80% of SLM was released within 4 h and after 10 h the percentage reached 100%. In the case of the micellar formulations, no SLM was released in GF and IF, confirming that the PEG chains in the shell of the micelles protect the core containing SLM from the gastric acid and intestinal fluids. This represents a promising result because we can assert that nanomicelles avoid the SLM degradation in the gastrointestinal tract and could improve the bioavailability of the extract. After 72 h in PBS, the cumulative release of SLM from PNM and MNM was only 3.2 ± 0.1% and 3.4 ± 0.2%, respectively, indicating that TPGS did not influence the release properties of the nanomicelles. The slow release of SLM observed for PNM and MNM might be due to the strong hydrophobic interaction between the extract and the inner core of the nanomicelles. Thus, both PNM and MNM are able to extend the residence time of SLM in vivo. At the end of the experiments, the samples in the dialysis bags were analyzed by DLS to assess the physical stability of SLM-PNM and SLM-MNM.

### 2.6. Parallel Artificial Membrane Permeability Assay (PAMPA)

The Parallel Artificial Membrane Permeability Assay (PAMPA) permits the fast in vitro determination of the ability of a compound to permeate artificial membranes by passive diffusion and therefore to estimate the gastrointestinal absorption after oral administration [51]. PAMPA gives information not only on the permeability of single molecules, but also on the behavior of the extracts, and recently the test was introduced to study formulated drugs [24,26,52,53,54,55]. In this work, PAMPA was used to evaluate to effect of nanomicelles on SLM permeability.

**Figure 4:**
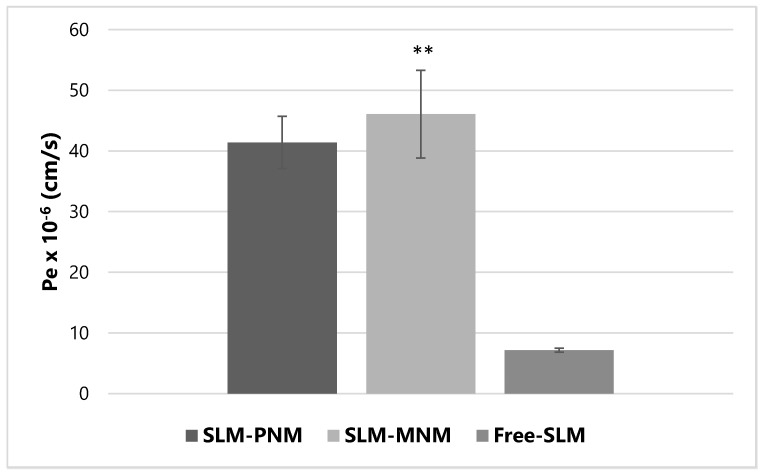
Effective permeability (P_e_) of free-silymarin (Free-SLM), silymarin-loaded polymeric nanomicelles (SLM-PNM) and silymarin-loaded mixed nanomicelles (SLM-MNM). Data are expressed as mean ± SD, *n* = 3. ** *p* < 0.01 vs. free silymarin, by Kruskal-Wallis test and Dunn’s multiple comparisons test.

The results displayed in Figure 4 indicate that both PNM and MNM increased the effective permeability (Pe) of SLM. Statistical analysis confirmed a significant improvement of permeability coefficient of SLM when formulated into MNM (** *p* < 0.01 vs. free-SLM) and, borderline, into PNM (*p* = 0.069; Kruskal–Wallis test and Dunn’s multiple comparisons test). The increase in SLM permeation when formulated into the nanomicelles is attributable to the increased lipophilicity of the extract [56]. Mass balance was higher than 80% for all the experiments, indicating that the calculated Pe is useful for in vitro prediction [57].

However, PAMPA lacks pore-mediated permeability and transporters, so it is not suitable to evaluate the influence of TPGS on P-gp mediated efflux on SLM. For this reason, permeation studies with a biological layer based on Caco-2 cells were also performed.

### 2.7. Caco-2 Experiments

The Caco-2 cell line is considered a viable model to estimate human intestinal absorption [55]. In the present work, the effect of the different dilutions (from 2-times to 100-times) of nanomicelles on the cell viability was investigated during 24 h to select the optimal concentration and time of exposure for transport experiments. Both formulations showed very low cytotoxicity after 12 h of exposure at the tested concentrations, proven by a cell viability value of >80%. In addition, the Lucifer yellow passage was less than 3%, indicating the integrity of the layer [24]. However, a notable reduction of the cell viability (>50%) compared to untreated control cells was evidenced after 24 h of incubation with formulations diluted 2-times. Thus, considering that a cell viability ≥80% is required for acceptable in vitro estimation and to allow the detection of the permeated SLM by HPLC-DAD analyses, both formulations were diluted two times and the duration of the transport experiments was set to 12 h.

Since SLM is a substrate of P-gp [36], the absorption (AP-BL) and efflux (BL-AP) of SLM were investigated adding SLM-PNM, SLM-MNM and free-SLM to the Transwell upper chamber (AP) for the absorption study and to the lower chamber (BL) for the efflux study (Table 7).

**Figure 5:**
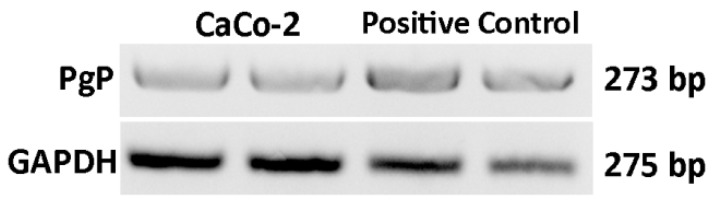
Representative analysis of P-gp expression in Caco-2 cells. PCR products were separated on 1.8% agarose gel containing Safeview.

The expression of P-gp gene in our Caco-2 cells was verified by RT-PCR. As shown in Figure 5, the cells used in the present experiment showed expression levels comparable to that of positive control (HCT-8 cancer cell line) [58].

MNM had a significant higher apparent permeability (P_app_) compared with PNM in AP-BL experiments. Meanwhile, free-SLM was not detected into the basolateral chamber. These results are attributable to the presence of TPGS, which increases not only the solubility of SLM but also its permeation across the intestinal epithelium, amplifying the effect of Soluplus [38]. To investigate the influence of Soluplus and TPGS on P-gp efflux, the secretory permeability studies were also performed. In this case, the Papp value of free SLM was ~6-fold higher compared with SLM-PNM and SLM-MNM, suggesting that ATP-dependent intestinal transporters, such as the P-gp proteins, mediate the efflux of SLM and that nanomicelles have a significant inhibitory effect on these transporters. Moreover, the efflux ratio for SLM-MNM was significantly lower compared to SLM-PNM, indicating that the combination of Soluplus and TPGS might play a synergistic effect in the intestinal absorption of SLM. These results confirmed that the developed MNM represent a promising approach for optimizing the performance of nanomicelles as drug delivery systems.

The mechanisms involved in the internalization of PNM and MNM were explored by performing uptake experiments in the presence of an energy depletion agent (sodium azide), a clathrin-dependent endocytosis inhibitor (chlorpromazine) or a caveolin-dependent endocytosis inhibitor (indomethacin) or by setting the temperature at 4 °C to inhibit active uptake processes [24]. These studies were performed for 1 h, since the blocking of one uptake pathway may result in activation of other endocytic mechanisms, which might confound the interpretation of the data [59].

**Figure 6:**
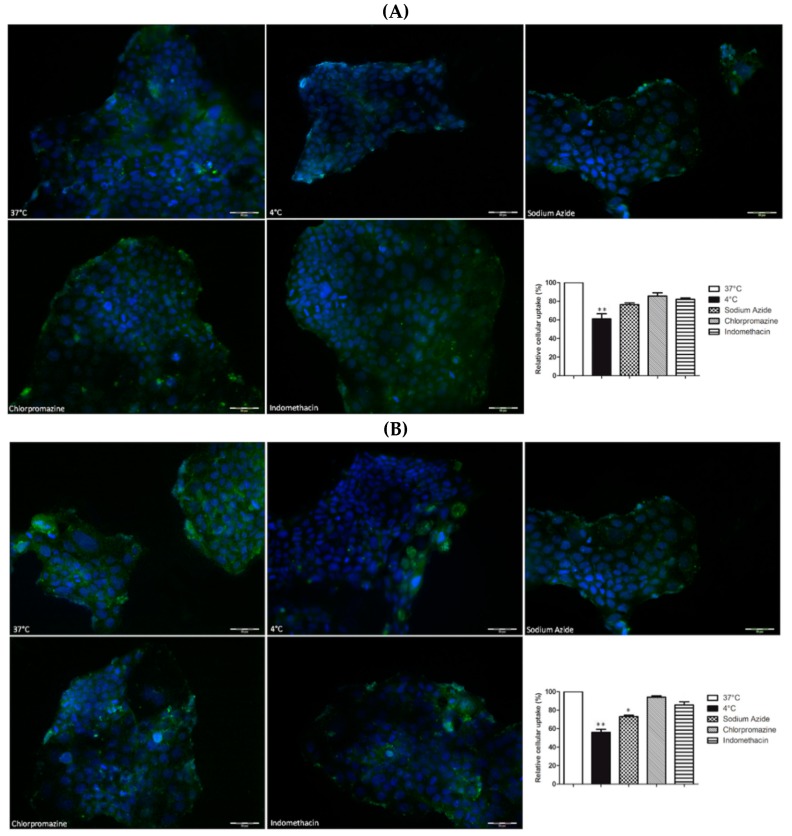
Cellular uptake of FITC-PNM (**A**) and FITC-MNM (**B**), green staining, in Caco-2 cells after 1 h of exposure at 37 °C (100%), at 4 °C or in the presence of endocytic inhibitors. Nuclei stained with DAPI. Final magnification 20×, scale bar 50 µm. Results are expressed as mean ± SEM, *n* = 3. ** *p* < 0.01 vs. 37 °C; * *p* < 0.05 vs. 37 °C, by Kruskal-Wallis test and Dunn’s multiple comparisons test.

As evidenced in Figure 6, the cellular uptake of both FITC-PNM and FITC-MNM was statistically inhibited at 4 °C (** *p* < 0.01 vs. 37 °C by Kruskal-Wallis test) and in the presence of sodium azide only for the MNM formulation (* *p* < 0.05 vs. 37 °C by Kruskal-Wallis test). Meanwhile, the presence of indomethacin or chlorpromazine slightly affected the internalization of FITC-loaded nanomicelles. These data suggested that both formulations entered into Caco-2 cells via energy-dependent mechanisms, in agreement with previously published data with similar formulations [47].

### 2.8. DPPH Assay

The antioxidant activity plays a key role in SLM therapeutic properties [2,3]. In this work, the DPPH radical scavenging activity of free-SLM, SLM-loaded nanomicelles and empty formulations was determined to investigate the effect of PNM and MNM on the antioxidant properties of SLM.

**Figure 7:**
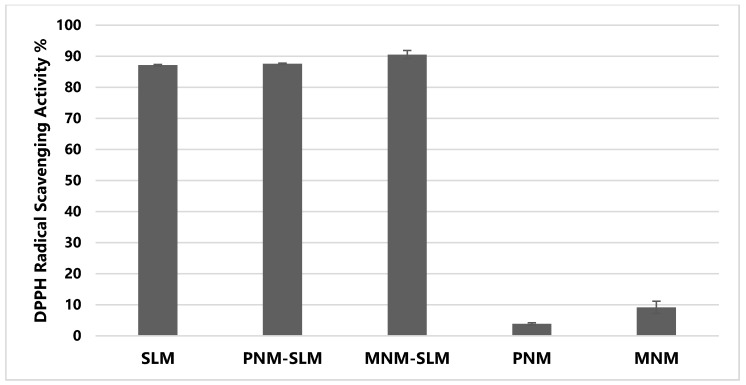
Antioxidant activity of SLM in solution, SLM-loaded polymeric nanomicelles (SLM-PNM), SLM-loaded mixed nanomicelles (SLM-MNM), empty polymeric nanomicelles (PNM) and empty mixed nanomicelles (MNM) (Mean ± SD, *n* = 3).

As reported in Figure 7, SLM showed a strong antioxidant activity, since the DPPH radical scavenging activity was ~90%. Moreover, it is possible to note that both nanomicellar formulations did not reduce the DPPH inhibition property of the extract. In particular, SLM loading into MNM determined a slight increase in the percentage of the radical scavenging activity. This might be due to the presence of TPGS in MNM, which is characterized by a moderate antioxidant activity [60,61], as also confirmed by different antioxidant properties of the empty nanomicelles.

## 3. Materials and Methods

### 3.1. Materials

Silymarin powder (SLM, ≥30% silybin (silybin A + silybin B) was obtained from Sigma Aldrich (https://www.sigmaaldrich.com/catalog/DataSheetPage.do?brandKey=SIGMA&symbol=S0292). The other compounds identified were the flavonolignans silychristin, silydianin, isosilybin A and isosilybin B and the flavanonol taxifolin. The extract title was 54.3% according to our previous work [24]), d-α-Tocopherol polyethylene glycol 1000 succinate (TPGS), silibinin (≥98%, HPLC), fluorescein 5(6)-isothiocyanate (FITC, ≥90%, HPLC), pepsin, bile salts, pancreatic lipase, pancreatin, phosphate buffered saline BioPerformance Certified pH 7.4 (PBS), Tween^®^80, lecithin (≥99%, TLC) lyophilized powder, cholesterol BioReagent (≥99%), 1,1-Diphenyl-2-picrylhydrazyl radical (DPPH), human serum albumin (≥96%, HSA) lyophilized powder and all analytical grade and HPLC grade solvents were obtained from Sigma Aldrich (Saint Louis, MO, USA) with the support of Sigma Aldrich Italia (Milan, Italy). Soluplus^®^ was a gift from BASF (Ludwigshafen, Germany) with the support of BASF Italia, BTC Chemical Distribution Unit (Cesano Maderno, Monza e Brianza, Italy). Distilled water was obtained from a Simplicity^®^UV Water Purification System, Merck Millipore (Darmstadt, Germany).

### 3.2. Methods

#### 3.2.1. Nanomicelles Fabrication

SLM-PNM and SLM-MNM were prepared by the thin film method [39]. Appropriate amounts of Soluplus, TPGS (only for MNM) and SLM were dissolved in 20 mL MeOH/CH_2_Cl_2_ mixture (80:20 *v/v*). Then, the solvents were evaporated at 30 °C by a rotary evaporator for 30 min until the formation of a thin film. Finally, the film was hydrated with 5 mL of distilled water under sonication for 5 min followed by 20 min of magnetic stirring at 300 rpm.

Empty PNM and MNM were prepared by the same method. Fluorescent formulations were obtained adding FITC instead of SLM. The total polymer concentration for both PNM and MNM was 5% *w/v*. The composition of the developed formulations is reported in Table 8.

#### 3.2.2. Theoretical Critical Micellar Concentration (CMC)

The theoretical CMC (*CMC_theor_*) value for PNM and MNM was calculated using the following equation [45,62],

(1) $$\frac{1}{CMC_{theor}}=\frac{X_{Soluplus}}{CMC_{Soluplus}}+\frac{X_{TPGS}}{CMC_{TPGS}}$$

where $X_{\mathrm{Soluplus}}$ and $X_{\mathrm{TPGS}}$ are the molar fractions of Soluplus and TPGS (considered only for MNM), and *CMC_Soluplus_* and *CMC_TPGS_* are the CMC values of Soluplus and TPGS, respectively.

The molar fractions of Soluplus and TPGS (only for MNM) were calculated by the ratio between the moles of the constituent and the total moles of the constituents of the mixture.

#### 3.2.3. Determination of Solubilization Capacity

The solubilization properties of PNM and MNM were investigated adding an excess of SLM to 5 mL of empty micellar solutions in sealed glass bottles, which were then kept under magnetic stirring at room temperature. After 24 h, the samples were centrifuged at 14,000 rpm for 10 min, and SLM concentration in the supernatants was determined by HPLC after proper dilution with MeOH [63]. Then, the solubility factor (*S_f_*) was calculated according the equation,

(2) $$S_{f}=\frac{S_{mic}}{S_{w}}$$

where *S_mic_* is the solubility of SLM in each micellar formulation and *S_w_* is the water solubility of the extract. SLM analyses were performed, employing an HP 1100 Liquid Chromatograph (Agilent Technologies, Santa Clara, CA, USA) equipped with a UV detector and a Luna Omega Polar (150 mm × 3 mm, 5 µm) (all from Agilent Technologies) RP-C18 analytical column. The software was HP 9000 (Agilent Technologies). SLM detection was at a wavelength of 288 nm. The mobile phase consisted of: (A) formic acid/water pH 3.2, (B) acetonitrile and (C) methanol. The following gradient profile was applied: 0–2 min 10% B and 10% C, 2–6 min 15% B and 22% C, 6–11 min 20% B and 30% C, 11–16 min 30% B and 40% C, 16–18 min 30% B and 40% C, 18–20 min 40% B and 40% C, 20–23 min 40% B and 40% C, 23–27 min 10% B and 10% C. The flow rate was 0.5 mL/min. The calibration curve was prepared using standard silibinin dissolved in methanol from a concentration range of 0.001–0.100 μg/μL, and the concentration absorption relationship was above 0.999.

#### 3.2.4. Nanomicelles’ Physical and Morphological Characterization

The average hydrodynamic diameter and the size distribution of nanomicelles (polydispersity index, PdI) were determined by dynamic light scattering (DLS, Zsizer Nanoseries ZS90, Malvern Instrument, Worcestershire, UK) at 25 °C and at a scattering angle of 90°. The zeta potential was evaluated measuring the electrophoretic mobility of nanomicelles by electrophoretic light scattering technique (ELS) employing the same instrument. The results were expressed as the average of three measurements.

The morphology of PNM and MNM was investigated by means of transmission electron microscopy (TEM, Jeol 1010, Tokyo, Japan). Before the analyses, the samples were placed onto a 200-mesh copper grid coated with carbon and negative stained with 1% *w/v* phosphotungstic acid solution (Electron Microscopy Sciences, Hatfield, PA, USA) [64].

#### 3.2.5. Drug Loading and Encapsulation Efficiency

The drug loading (DL%) and encapsulation efficiency (EE%) were determined by membrane filtration method [65]. SLM-PNM, SLM-MNM, FITC-PNM and FITC-MNM were filtered with a 0.45 μm filter membrane. Non-encapsulated SLM (or FITC) was retained on the membrane, while 20 μL of the filtrate was disrupted with 980 μL of MeOH. The amount of SLM (or FITC) encapsulated and loaded in the nanomicelles was quantified by HPLC. SLM analyses were carried out as described above, FITC was quantified employing the same instrument and apparatus, while the mobile phase consisted of (A) formic acid/water pH 3.2 and (B) acetonitrile. The flow rate was set at 0.5 mL/min. The gradient profile was: 0–2 min 20% B, 2–22 min 20–85% B, 22–25 min 85–100% B, 25–28 min 20% B. FITC chromatograms were acquired at a wavelength of 224 nm. For the calibration curve, different concentrations ranging from 0.002 μg/μL to 0.057 μg/μL were used. The linear correlation coefficient was >0.999.

The DL% and EE% were calculated by following equations:

(3) $$DL\%=\frac{Weight\;of\;SLM\;\left(or\;FITC\right)\;in\;nanomicelles}{Weight\;of\;SLM\;\left(or\;FITC\right)\;fed\;+Weight\;of\;the\;excipients}\;\times \;100$$

(4) $$EE\%=\frac{Weight\;of\;SLM\;\left(or\;FITC\right)in\;nanomicelles}{Weight\;of\;SLM\;\left(or\;FITC\right)fed\;}\times 100$$

All samples were analyzed in triplicate.

#### 3.2.6. Cloud Point

The cloud point value of SLM-PNM and SLM-MNM was determined by immersing glass tubes containing 4 mL of micellar formulations in a water bath at room temperature. Then, the temperature was increased until the appearance of the samples changed from clear to turbid. After that, the micellar formulations were cooled down, and the measurements were replicated to obtain a triplicate [44,66].

#### 3.2.7. Stability Studies

##### Storage Stability Studies

To investigate the physical and chemical stability of SLM-PNM and SLM-MNM, the samples were transferred into glass bottles sealed with plastic caps and stored both at room temperature and 4 °C over a period of three months. The average diameter, PdI, zeta potential, EE% and any SLM precipitation phenomena were evaluated.

##### Gastrointestinal Stability Studies

The physical stability of SLM-loaded nanomicelles was evaluated after incubation of formulations in simulated gastric fluid (GF) followed by simulated intestinal fluid (IF). GF consisted of 2 g of NaCl, 3.2 g of pepsin and 7 mL of HCl diluted to 1 L. The pH value was adjusted to 1.2 with HCl 1 N. SLM-PNM and SLM-MNM were mixed with GF (final ratio 1:1 *v*/*v*) and maintained at 37 °C under continuous shaking (250 rpm) for 2 h. Then, the digested samples were incubated for 6 h at 37 °C with IF. IF consisted of pancreatic lipase (4.8 mg/mL), bile salts (5 mg/mL), pancreatin (0.5 mg/mL) and CaCl_2_ 750 mM. The pH value was adjusted to 7.0 with NaOH 1 N. After the incubation both in GF and IF, the average diameter and PdI were evaluated [53,67]. The studies were performed in triplicate.

##### Stability in Blood Conditions

The average diameter and size distribution of SLM-PNM and SLM-MNM were evaluated after 72 h of incubation in PBS (pH 7.4) in the absence or in the presence of human serum albumin at a physiological concentration (HSA, 45 mg/mL). The samples were diluted into the media to obtain a polymer concentration of 0.5 mg/mL, then were incubated for 72 h at 37 °C under shaking (250 rpm). At scheduled time points (24 h, 48 h, 72 h), aliquots of the samples were collected for DLS analyses [68,69]. The assays were performed in triplicate.

#### 3.2.8. In Vitro Release Studies

SLM release from PNM and MNM was studied by the dialysis bag method. In brief, 2 mL of SLM-PNM, SLM-MNM and SLM solution with equivalent SLM concentration were added to a regenerated cellulose dialysis membrane (Spectrum Laboratories, Inc., Breda, The Netherlands, MWCO 12–14 kD) and then immersed in 200 mL of the release media at 37 °C under magnetic stirring at 100 rpm. Enzyme-free GF, IF and PBS with 0.5% Tween 80 were selected as release media. SLM release in GF was monitored during 2 h, in IF over a period of 6 h, while in PBS during 72 h. At predetermined time intervals, 1 mL of release medium was withdrawn for HPLC analyses and replaced with the same volume of fresh release medium. All experiments were performed in triplicate.

#### 3.2.9. PAMPA Studies

PAMPA was carried out on 96-well filter plates (Millipore, Billerica, MA, USA). A lecithin (1% *w*/*v*) and cholesterol (0.8% *w*/*v*) solution in 1,7-octadiene was prepared as artificial membrane. Then, 10 μL of this solution was added on the PVDF membrane filters in the donor compartments. After the application of the artificial membrane, 0.25 mL of SLM-PNM, SLM-MNM and SLM solution was added to each well of the donor compartment, and 0.25 mL of 5% DMSO solution in PBS was added to each well acceptor compartment. Then, after placing the donor compartment into the acceptor compartment, the system was transferred into a sealed container and incubated at room temperature for 2 h. After the incubation, SLM concentration in the donor and acceptor compartment was determined by HPLC [24,26,52,53,55,56]. The effective permeability (*P_e_*, cm/s) was estimated with the following equation,

(5) $$P_{e}=\frac{-ln\left[1-\frac{C_{At}}{C_{eq}}\right]}{A\left(\frac{1}{V_{D}}+\frac{1}{V_{A}}\right)t}$$

where *A* is the active surface area, *V_D_* and *V_A_* the well volume of the donor and acceptor plate, respectively, *t* the incubation time (s), *C_At_* and *C_Dt_* the concentration of SLM in the acceptor and donor plate at time *t*, respectively. C_eq_ was calculated according to:

(6) $$C_{eq}=\frac{\left[C_{Dt}\times V_{D}+C_{At}\times V_{A}\right]}{V_{A}+V_{D}}$$

Mass balance was also calculated. The assay was performed in triplicate.

#### 3.2.10. Caco-2 Cell Lines

The colorectal adenocarcinoma cell line (Caco-2) was purchased from American Tissue Type Culture Collection (Manassas, VA, USA) and cultured in Dulbecco’s modified Eagle’s medium (Thermo Fisher Scientific, Rodano, Milan, Italy) with 20% fetal bovine serum (FBS) (Thermo Fisher Scientific, Rodano, Milan, Italy), 100 U/mL penicillin-streptomycin (Thermo Fisher Scientific, Rodano, Milan, Italy) in 5% CO2 at 37 °C. An MTS assay was used to determine the cell viability after exposure to of MNM, PNM, SLM-MNM and SLM-PNM or to free-SLM for 12 h [70]. The relative cell viability was expressed as a percentage of the untreated control group.

##### Transport Studies

Caco-2 cells were seeded into 12-well PET Transwell plates (1.13 cm^2^ growth surface area and pore size 0.4 µm, Greiner Bio-One, Milan, Italy) at a density of 2 × 10^5^ cells/cm^2^ and grown for 21 days to form a confluent monolayer. Before the transport studies, the integrity of the cellular barrier was assessed using Lucifer yellow (LY) permeability test [56]. Absorptive (AP-BL) and secretive (BL-AP) transport experiments in the Caco-2 cell monolayer were performed by incubating SLM-MNM, SLM-PNM (diluted 2 times) and SLM aqueous solution for 12 h in the apical or in the basal compartment. Samples of media collected from the basal or apical compartment were used to detect SLM by HPLC. The apparent permeability (*P_app_*, cm/s) was calculated with the following equation [24],

(7) $$P_{app}=V_{A}/\left(A\cdot C_{D_{0}}\right)\times \left(\Delta C_{A}/\Delta t\right)$$

where *V_A_* is the acceptor volume (mL), *A* the surface area (cm^2^), *C_D0_* the concentration in the donor chamber at start of experiment and $\Delta C_{A}/\Delta t$. the change in concentration in the acceptor compartment over time (s).

The efflux ratio was determined according to:

(8) $$Efflux\;ratio\;=\;\frac{P_{app}\left(BL-AP\right)}{P_{app}\left(AP-BL\right)}$$

##### Uptake Studies

To identify the uptake mechanism of the developed nanomicelles, Caco-2 cells were pre-incubated with sodium azide (an ATP synthesis inhibitor, 1 µM), chlorpromazine (a clathrin blocker, 15 µM) and indomethacin (a caveolin-dependent endocytosis inhibitor, 25 µM) for 30 min followed by the addition of FITC-PNM or FITC-MNM 1:10 for 1 h and maintained at 4 °C during the exposure. At the end of the treatments, the amount of FITC was quantified on cellular lysate by HPLC analyses. In parallel, Caco-2 cells, grown on histological slides, were treated in the same conditions, fixed in 4% formaldehyde in 0.1 mol/L PBS pH 7.4, for 10 min and observed by fluorescence microscopy (Labophot-2 Nikon, Tokyo, Japan). Ten photomicrographs were randomly taken for each sample, and fluorescence was measured using ImageJ 1.33 image analysis software (http://rsb.info.nih.gov/ij).

##### RT-PCR

Total RNA was extracted from cell lysates using the Nucleo Spin^®^ RNA kit (Macherey-Nagel, Bethlehem, USA) according to manufacturer’s instructions. The P-gp gene expression in Caco-2 cells was evaluated by RT-PCR analysis (Table 9), as previously described [54].

#### 3.2.11. Antioxidant Activity Studies

The antioxidant activity of SLM was assayed by the DPPH (1,1-diphenyl-2-picrylhydrazyl) test.

SLM-PNM, SLM-MNM, and SLM solution were diluted with ethanol to obtain an equivalent concentration of SLM, then 1 mL of each sample was added to an equal volume of DPPH ethanolic solution (100 μM) and incubated in the dark for 20 min at room temperature. The antioxidant activity of empty PNM and MNM was also checked. The absorbance of the solutions was measured at 517 nm against blank (ethanol) by a spectrophotometer [71].

The antioxidant activity of SLM (or free radical scavenging activity) was calculated according to the following formula,

(9) $$Antioxidant\;activity\%=\frac{\left[\left(A_{DPPH}-\;A_{sample}\right)\right]}{A_{DPPH}}\;\times \;100$$

where *A_DPPH_* is the control (absorbance of DPPH radicals without sample) and *A_sample_* is the absorbance of radicals after reacting with the sample. The experiments were performed in triplicate.

#### 3.2.12. Statistical Analysis

Data were analyzed by Kruskal-Wallis test and Dunn’s multiple comparisons test or by using Mann Whitney test and expressed as mean ± standard error (SEM) or mean ± standard deviation (SD) of three independent experiments. All analyses were carried out using GraphPad Prism 7.0 (GraphPad Software, San Diego, CA, USA). P value of 0.05 was considered significant.

## 4. Conclusions

Since the clinical use of SLM is limited by its poor oral bioavailability, in this study the effect of Soluplus PNM and Soluplus/TPGS MNM on intestinal absorption and secretion was investigated. The developed formulations showed small particle size (~50 nm), narrow PdI (~0.1), high encapsulation efficiency (>92%) and did not interfere with the antioxidant capacity of the extract. PNM and MNM increased the aqueous solubility of SLM by ~6 times and exhibited proper CMC for oral administration. The stability during storage over three months and in simulated physiological conditions was assessed. PAMPA demonstrated that PNM and MNM enhanced the passive diffusion of SLM. Based on the cellular results, nanomicelles have a significant inhibitory effect on the P-gp-mediated efflux of SLM, and MNM increased SLM permeability through Caco-2 cells’ monolayer compared to unformulated extract and PNM. The obtained results encourage further studies on silymarin-loaded nanomicelles as oral formulation.

## Figures and Tables

**Table 1:** Physical and chemical characterization of empty, SLM- and fluorescein isothiocyanate (FITC)-loaded PNM and MNM (Mean ± SD, *n* = 3).

Sample	Average Diameter (nm)	PdI	Zeta Potential (mV)	EE%	LC%
PNM	59.7 ± 0.1	0.06 ± 0.02	−5.5 ± 0.6	-	-
MNM	60.2 ± 2.5	0.05 ± 0.01	−4.7 ± 0.6	-	-
SLM-PNM	61.3 ± 6.0	0.10 ± 0.03	−4.7 ± 0.5	93.0 ± 3.9	2.9 ± 0.2
SLM-MNM	61.5 ± 4.3	0.10 ± 0.03	−4.3 ± 0.5	92.9 ± 5.3	2.9 ± 0.2
FITC-PNM	60.6 ± 2.1	0.05 ± 0.01	−6.2 ± 0.3	95.3 ± 1.2	1.9 ± 0.1
FITC-MNM	63.3 ± 1.4	0.10 ± 0.01	−5.9 ± 0.2	95.7 ± 1.2	1.9 ± 0.0

PNM: Polymeric nanomicelles; MNM: Mixed nanomicelles; SLM-PNM: Silymarin-loaded polymeric nanomicelles; SLM-MNM: Silymarin-loaded mixed nanomicelles; FITC-PNM: Fluorescein isothiocyanate polymeric nanomicelles; FITC-MNM: Fluorescein isothiocyanate mixed nanomicelles.

**Table 2:** SLM solubility and solubility factor in PNM, MNM and water at room temperature (Mean ± SD, *n* = 3).

Sample	SLM Solubility (mg/mL)	S_f_
Soluplus	2.41 ± 0.03	6.51
Soluplus/TPGS 20:1	2.05 ± 0.07	5.54
Soluplus/TPGS 10:1	1.89 ± 0.01	5.11
Soluplus/TPGS 5:1	1.83 ± 0.04	4.95
Soluplus/TPGS 4:1	1.78 ± 0.04	4.81
Soluplus/TPGS 3:1	1.64 ± 0.04	4.43
Soluplus/TPGS 2:1	1.58 ± 0.04	4.27
Water	0.37 ± 0.01	-

SLM: Silymarin; S_f_: Solubility factor.

**Table 3:** Storage stability test of SLM-PNM and SLM-MNM at 4 °C (Mean ± SD, *n* = 3).

Sample	Average Diameter (nm)	PdI	Zeta Potential (mV)	EE%	SLM Precipitate
SLM-PNM	57.5 ± 0.5	0.07 ± 0.01	−4.3 ± 0.2	93.2 ± 0.1	NO
SLM-MNM	59.7 ± 0.1	0.04 ± 0.01	−4.5 ± 0.5	92.7 ± 0.1	NO

SLM-PNM: Silymarin-loaded polymeric nanomicelles; SLM-MNM: Silymarin-loaded mixed nanomicelles; PdI: polydispersity index; EE%: encapsulation efficiency.

**Table 4:** Storage stability test of SLM-PNM and SLM-MNM at room temperature (Mean ± SD, *n* = 3).

Sample	Average Diameter (nm)	PdI	Zeta Potential (mV)	EE%	SLM Precipitate
SLM-PNM	56.7 ± 0.2	0.06 ± 0.01	−4.4 ± 0.2	92.7 ± 0.1	NO
SLM-MNM	57.8 ± 0.2	0.06 ± 0.01	−4.7 ± 0.4	92.8 ± 0.1	NO

SLM-PNM: silymarin-loaded polymeric nanomicelles; SLM-MNM: silymarin-loaded mixed nanomicelles; PdI: polydispersity index; EE%: encapsulation efficiency.

**Table 5:** Physical stability of SLM-loaded nanomicelles in simulated gastric fluid (GF) and simulated intestinal fluid (IF) (Mean ± SD, *n* = 3).

	GF		IF	
Sample	Average Diameter (nm)	PdI	Average Diameter (nm)	PdI
SLM-PNM	58.7 ± 1.1	0.12 ± 0.01	65.4 ± 2.2	0.20 ± 0.02
SLM-MNM	61.3 ± 0.8	0.11 ± 0.01	65.3 ± 1.4	0.13 ± 0.01

SLM-PNM: silymarin-loaded polymeric nanomicelles; SLM-MNM: silymarin-loaded mixed nanomicelles; GF: simulated gastric fluid; IF: simulated intestinal fluid; PdI: polydispersity index.

**Table 6:** Physical stability of SLM-loaded nanomicelles in phosphate buffer saline (PBS) without and with HSA (Mean ± SD, *n* = 3).

SLM-PNM			SLM-MNM	
Medium	Average Diameter (nm)	PdI	Average Diameter (nm)	PdI
PBS 24 h	68.0 ± 1.1	0.08 ± 0.01	69.7 ± 3.4	0.07 ± 0.01
PBS 48 h	64.1 ± 1.8	0.12 ± 0.02	75.8 ± 4.4	0.08 ± 0.03
PBS 72 h	66.6 ± 1.2	0.09 ± 0.01	72.0 ± 1.4	0.11 ± 0.01
PBS + HSA 24 h	69.6 ± 1.3	0.21 ± 0.02	75.6 ± 6.3	0.26 ± 0.01
PBS + HSA 48 h	70.6 ± 0.4	0.24 ± 0.01	74.0 ± 2.1	0.24 ± 0.01
PBS + HSA 72 h	70.9 ± 0.2	0.25 ± 0.01	70.2 ± 1.1	0.25 ± 0.01

SLM-PNM: silymarin-loaded polymeric nanomicelles; SLM-MNM: silymarin-loaded mixed nanomicelles; PBS: phosphate buffer saline; HSA: human serum albumin; PdI: polydispersity index.

**Table 7:** Apparent permeability coefficients (P_app_) of free-SLM and SLM-loaded nanomicelles. (Mean ± SD, *n* = 3).

Sample	P_app_ × 10^−9^ (cm/s) AP-BL	P_app_ × 10^−9^ (cm/s) BL-AP	Efflux Ratio
SLM-PNM	9.10 ± 0.14	6.11 ± 1.25	0.67 ± 0.15
SLM-MNM	140.90 ± 15.06 *	6.16 ± 1.75	0.04 ± 0.01 *
Free-SLM	N.D.	17.29 ± 0.30	N.A.

SLM-PNM: Silymarin-loaded polymeric nanomicelles; SLM-MNM: Silymarin-loaded mixed nanomicelles; Free-SLM: Silymarin aqueous solution; N.D.: Not detected; N.A.: Not applicable. * *p* < 0.05 vs. SLM-PNM by Mann Whitney test.

**Table 8:** Nanomicelles composition.

Sample	Soluplus (mg)	TPGS (mg)	SLM (mg)	FITC (mg)
PNM	250	-	-	-
MNM	238	12	-	-
SLM-PNM	250	-	15	-
SLM-MNM	238	12	15	-
FITC-PNM	250	-	-	5
FITC-MNM	238	12	-	5

PNM: Polymeric nanomicelles; MNM: Mixed nanomicelles; SLM-PNM: Silymarin-loaded polymeric nanomicelles; SLM-MNM: Silymarin-loaded mixed nanomicelles; FITC-PNM: Fluorescein isothiocyanate polymeric nanomicelles; FITC-MNM: Fluorescein isothiocyanate mixed nanomicelles.

**Table 9:** Primers sequences.

Gene	Primer Forward	Primer Reverse	Size
P-gp	CAGAGGCTCTATGACCCCAC	CAACTGGGCCCCTCTCTCTC	273
GAPDH	CCCTCAAGGGCATCCTGGGCT	GCAGGGACTCCCCAGCAGTGA	275

P-gp: P-glycoprotein; GAPDH: Glyceraldehyde 3-phosphate dehydrogenase.
